# Supplementary material for: The Systems Biology Research Tool: evolvable open-source software
Source: BMC Syst Biol. 2008 Jun 29;2:55. doi: 10.1186/1752-0509-2-55 (PMC2446383; doi:10.1186/1752-0509-2-55)
Supplement: Additional file 1 — SBRT Archive. An archive of the current version of the Systems Biology Research Tool. [file 1752-0509-2-55-S1.zip › sbrt-1.4.0/doc/developers_guide/api/sbrt/shell/io/SerializedMapInputFile.html]

SerializedMapInputFile


|  |  |  |
| --- | --- | --- |
| **Overview** \| **Package** \| **Class** \| **Use** \| **Tree** \| **Deprecated** \| **Index** \| **Help** | |  |
| **PREV CLASS**   **NEXT CLASS** | **FRAMES**    **NO FRAMES**     **All Classes** |  |
| SUMMARY: NESTED \| FIELD \| CONSTR \| METHOD | DETAIL: FIELD \| CONSTR \| METHOD |  |


---


## sbrt.shell.io Class SerializedMapInputFile<K,V>

```
java.lang.Object
  sbrt.shell.io.SerializedInputFile<java.util.Map<K,V>>
      sbrt.shell.io.SerializedMapInputFile<K,V>
```

**Type Parameters:**: `K` - the key type.: `V` - the value type.

**All Implemented Interfaces:**: java.lang.Iterable<java.util.Map<K,V>>, InputFile<java.util.Map<K,V>>, MapInputFile<K,V>, SbrtFile

---

``` public class SerializedMapInputFile<K,V> extends SerializedInputFile<java.util.Map<K,V>> implements MapInputFile<K,V> ```

This class is used to read input files containing serialized
maps.

**Author:**
:   This class was written and documented by
    Jeremiah Wright while in the Wagner lab.

---

**Constructor Summary**

`SerializedMapInputFile(java.lang.String fileName,
FileFormat fileFormat)`
  
          Constructs a new input file using the provided arguments.

`SerializedMapInputFile(java.lang.String fileName,
FileFormat fileFormat,
java.util.Set<? extends K> requiredKeys)`
  
          Constructs a new input file using the provided arguments.


| **Method Summary** | |
| --- | --- |
| `java.util.Iterator<java.util.Map<K,V>>` | `iterator()`             Returns an iterator over this file. |

**Methods inherited from class sbrt.shell.io.SerializedInputFile**

`close, finalize, getCanonicalName, getFileFormat, getOriginalName, isClosed, isValidFormat, objectIterator`

**Methods inherited from class java.lang.Object**

`clone, equals, getClass, hashCode, notify, notifyAll, toString, wait, wait, wait`

**Methods inherited from interface sbrt.shell.io.SbrtFile**

`close, getCanonicalName, getOriginalName, isClosed`

**Constructor Detail**

### SerializedMapInputFile

```
public SerializedMapInputFile(java.lang.String fileName,
                              FileFormat fileFormat)
```

:   Constructs a new input file using the provided arguments.

    **Parameters:**: `fileName` - the name of the file to be read.: `fileFormat` - the format of the file to be read.

---


### SerializedMapInputFile

```
public SerializedMapInputFile(java.lang.String fileName,
                              FileFormat fileFormat,
                              java.util.Set<? extends K> requiredKeys)
```

:   Constructs a new input file using the provided arguments.

    **Parameters:**: `fileName` - the name of the file to be read.: `fileFormat` - the format of the file to be read.: `requiredKeys` - the keys that are required to be in the file.


**Method Detail**

### iterator

```
public java.util.Iterator<java.util.Map<K,V>> iterator()
```

:   Returns an iterator over this file.

    :   **Specified by:**: `iterator` in interface `java.lang.Iterable<java.util.Map<K,V>>` **Overrides:**: `iterator` in class `SerializedInputFile<java.util.Map<K,V>>`
    :   **Returns:**: an iterator over this file.


---


|  |  |  |
| --- | --- | --- |
| **Overview** \| **Package** \| **Class** \| **Use** \| **Tree** \| **Deprecated** \| **Index** \| **Help** | |  |
| **PREV CLASS**   **NEXT CLASS** | **FRAMES**    **NO FRAMES**     **All Classes** |  |
| SUMMARY: NESTED \| FIELD \| CONSTR \| METHOD | DETAIL: FIELD \| CONSTR \| METHOD |  |


---
